# Guidance for clinicians and patients with non-small cell lung cancer in the time of precision medicine

**DOI:** 10.3389/fonc.2023.1124167

**Published:** 2023-02-01

**Authors:** Liza C. Villaruz, Mark A. Socinski, Jared Weiss

**Affiliations:** ^1^ Hillman Cancer Center, Department of Medicine, Division of Hematology/Oncology, University of Pittsburgh Medical Center, Pittsburgh, PA, United States; ^2^ AdventHealth Cancer Institute, Orlando, FL, United States; ^3^ Division of Oncology, Lineberger Comprehensive Cancer at the University of North Carolina, Chapel Hill, NC, United States

**Keywords:** targeted therapy, non-small cell lung cancer, biomarker testing, patient education, next-generation sequencing

## Abstract

Major advances in the diagnosis and treatment of non-small cell lung cancer (NSCLC) have resulted in a sharp decline in associated mortality rates, thereby propelling NSCLC to the forefront of precision medicine. Current guidelines recommend upfront comprehensive molecular testing for all known and actionable driver alterations/biomarkers (*EGFR*, *ALK*, *ROS1*, *BRAF*, *KRAS*, *NTRK*, *MET*, *RET*, *HER2* [*ERBB2*], and PD-L1), especially in advanced disease stages, as they significantly influence response to therapy. In particular, hybrid capture-based next-generation sequencing (HC-NGS) with an RNA fusion panel to detect gene fusions is a veritable requirement at both diagnosis and progression (resistance) of any-stage non-squamous adenocarcinoma NSCLCs. This testing modality ensures selection of the most timely, appropriate, and personalized treatment, maximization of therapeutic efficacy, and prevention of use of suboptimal/contraindicated therapy. As a complement to clinical testing and treatment, patient, family, and caregiver education is also key to early screening and diagnosis, access to care, coping strategies, positive outcomes, and survival. The advent of social media and increased internet access has amplified the volume of educational and support resources, consequently changing the dynamics of patient care. This review provides guidance on integration of comprehensive genomic testing with an RNA fusion panel as a global diagnostic standard for all adenocarcinoma NSCLC disease stages and provides key information on patient and caregiver education and resources.

## 1 Introduction

In 2022, lung cancer remains the leading cause of cancer-related death in the United States (1). However, recent advances in early detection and treatment have caused a sharp decline in mortality rates, largely for non-small cell lung cancer (NSCLC) (1). Improved survival may reflect patients’ increased access to care, as earlier diagnosis significantly improves lung cancer outcomes, with a 5-year relative survival ranging from 6% for distant-stage disease to 60% for localized-stage disease (1, 2).

Constituting up to 85% of all lung cancer cases (3), NSCLCs are heterogenous, with diverse histologies and many oncogenic driver alterations that significantly influence response to standard therapy (4). Current guidelines recommend upfront molecular testing for all actionable biomarkers and driver alterations (see **Table 1**), especially in advanced disease stages (4, 11, 12). Major progress has been made in the management of NSCLC in the past decade, including identification and targeting of genetic abnormalities, which has propelled NSCLC to the forefront of precision medicine.

**Table 1:** Recommended Food and Drug Administration (FDA)-approved targeted therapies for treatment of known non-small cell lung cancer genetic alterations (4–9).

Gene	Alteration	Targeted Therapies	Line of Therapy
*EGFR*	Exon 19 deletion or exon 21 (L858R) substitution (with or without T790M)	Osimertinib (preferred/strongly recommended 1L option)	1L+
		Gefitinib	1L
		Erlotinib	1L
		Afatinib	1L
		Dacomitinib	1L
		Ramucirumab + Erlotinib	1L
	S768I, L861Q, and/or G719X	Afatinib	1L
	Exon 20 insertion (T790M substitution)	Amivantamab-vmjw	2L+
		Mobocertinib	2L+
*KRAS*	G12C	Sotorasib	2L+
		Adagrasib	2L+
*ALK*	Rearrangement (translocation)	Alectinib (preferred/strongly recommended 1L option)	1L+
		Brigatinib (preferred/strongly recommended 1L option)	1L+
		Lorlatinib (recommended 1L option)	1L+
		Ceritinib	1L+
		Crizotinib	1L
*ROS1*	Rearrangement (fusion)	Entrectinib	1L+
		Crizotinib	1L
*BRAF*	V600E	Dabrafenib	1L
		Dabrafenib + Trametinib	1L+
*NTRK1/2/3*	Fusion	Larotrectinib	1L+
		Entrectinib	1L+
*MET*	Exon 14 skipping mutation (METex14)	Capmatinib	1L+
		Tepotinib	1L+
*RET*	Rearrangement (fusion)	Selpercatinib	1L+
		Pralsetinib	1L+
*HER2 (ERBB2)*	Insertion	Fam-trastuzumab deruxtecan-nxki	2L+

For additional information on specific alterations and related therapeutics, see Chakravarty et al. (10) (oncokb.org).

Comprehensive genomic testing, including next-generation sequencing (NGS) with an RNA fusion panel, is a veritable requirement at both diagnosis and progression of non-squamous adenocarcinoma NSCLC (henceforth referred to as aNSCLC) to ensure selection of the most appropriate targeted therapies and improve outcomes. Despite the diagnostic necessity of NGS, real-world studies indicate that there is still a large proportion of patients with aNSCLC that do not undergo adequate genotyping at any stage of disease (13–15). Patient and caregiver education is also critical as education and resources can increase access to care and early screening and provide opportunities for broader support.

This review provides guidance on integration of comprehensive NGS with an RNA fusion panel into standard aNSCLC management practices as well as key information on patient, family, and caregiver education and resources to enhance patient participation in their health care journey. Because NGS is not routinely recommended for NSCLCs with squamous histology, our focus is on advanced non-squamous NSCLC with adenocarcinoma or other histology that presents features highly indicative of an oncogenic driver (4, 11, 12), noting that some guidelines recommend consideration of broad molecular testing for early-stage aNSCLC as well as advanced stages (16).

## 2 When to conduct NGS testing

In metastatic aNSCLC, it is vital to establish the correct histologic subtype and conduct comprehensive, parallel NGS at the time of initial diagnosis as there are a number of genomic alterations, each of which requires an associated molecular targeted therapy (**Table 1**) (4–8, 12). NGS with an appropriate fusion panel enables simultaneous detection of all existing biomarkers/alterations in any number of genes at any given timepoint and can detect rare molecular alterations with low prevalence/frequency (≤1%) (17) in samples with 20% or less malignant cells (12, 17), as recommended by experts (11). Guidelines strongly recommend comprehensive molecular profiling be performed before selecting therapy, if clinically feasible, to identify any driver alterations for which targeted therapies are already available, or to appropriately counsel patients regarding clinical trial enrollment (4, 11, 12). Availability of broad molecular genotyping results prior to initiation of first-line therapy is associated with significantly longer overall survival (18, 19).

In the event there is insufficient sample to allow testing for all currently recommended molecular drivers (see **Table 1**) (4, 11, 12), biopsy testing should be repeated. If retesting/re-biopsy is not possible, treatment should either be guided by available results or be dispensed as though driver oncogene alterations are not present (4).

If PD-L1 expression is elevated in patients with an oncogenic driver alteration, targeted therapy for the oncogenic driver should be given before immune checkpoint inhibitor treatment (4). Patients without a molecular target are treated with chemotherapy plus immunotherapy in most cases (4). However, if treatment is imperative prior to the receipt of molecular testing results, it is advisable to start with chemo(radio)therapy alone and hold on immunotherapy to reduce the risk of pneumonitis (20, 21). Clinicians must also consider overlapping toxicities between immune checkpoint inhibitors (ICIs) and targeted agents. In addition to the well-documented increase in risk of developing interstitial lung disease when osimertinib is administered after ICIs, others have shown potentially increased risk of high-grade skin and gastrointestinal adverse events when ICIs are combined with targeted agents (22) (although the scenario we are describing would be sequential use, the overlapping toxicities for these classes of agents are worth noting). Furthermore, many oncogenic drivers are negative predictors for immunotherapy efficacy, especially in the first-line setting. In first-line, targeted therapies yield higher response rates and are better tolerated than ICIs (4, 23–25). Given the potential impact on both patient safety and treatment efficacy, it is critical to obtain comprehensive molecular data before considering an immunotherapy-based approach.

In addition to initial diagnosis, comprehensive molecular testing should be employed to retest patients who have relapsed or become resistant to prior therapy. This step is essential to understanding resistance mechanisms and informing further treatment decisions (11, 26).

Although not the focus of this review, we note that NGS may be considered for earlier stages of disease due to the potential impact of identified *EGFR* and *ALK* alterations on selection of adjuvant and neoadjuvant treatment options (4, 16). However, tests focused on those alterations (eg, RT-PCR) could be considered in lieu of genome-wide testing in this setting.

## 3 Which sample type to test

While tissue-based NGS is currently the gold standard, plasma-based NGS is quickly becoming a routine part of clinical practice, and there are currently two FDA-approved plasma-based NGS assays commercially available (27, 28). NGS detection of circulating tumor (cell-free) DNA (ctDNA) alterations in plasma is a relatively noninvasive method for screening high-risk populations, guiding early diagnosis and treatment, monitoring relapse, and conducting prognostic evaluation (29). NILE trial results demonstrate that the real-world impact of ctDNA-based NGS on first-line treatment choice and patient outcomes in aNSCLC begins with a significant reduction in time to treatment initiation versus tissue-based NGS (30). The study confirmed that ctDNA analysis detected actionable mutations at a similar rate as tissue genotyping and similar response rates were achieved regardless of sample sources (30). However, plasma-based NGS is limited by the low abundance of ctDNA fragments in blood, which affects analytical sensitivity, with up to a 30% false-negative rate (4, 29). ctDNA assay results can also be obstructed by clonal hematopoietic and germline alterations (4, 29). Thus, guidelines recommend that ctDNA testing should not be used in lieu of a tissue-based diagnosis (4, 11, 12). ctDNA-only NGS should be considered in specific circumstances, such as in patients who are medically unfit for invasive tissue sampling and/or if there is insufficient tissue biopsy material for mutational analysis (4, 11, 12).

On the other hand, concurrent plasma- and tissue-based NGS should be used as a complementary approach to detect alterations and tumor burden in real time (31), thereby enabling monitoring of cancer recurrence and metastasis (29). In fact, guidelines suggest scheduling the biopsy concurrently with plasma testing referral (4). In the resistance/recurrence setting, plasma-based retesting is often preferable to tissue re-biopsy and supported by results from the NILE study (30). Real-world analyses of large NSCLC patient cohorts, including aNSCLC, have reported that plasma-based NGS increased the detection rate of clinically relevant oncogenic variants by as much as 65% versus tissue NGS alone (14, 32–34). Data support the major advantages of plasma-based testing to reduce time to treatment initiation and increase yield of targetable alteration detection (4). Not only is concurrent tissue- and plasma-based NGS clinically feasible, the significant increase in patients undergoing comprehensive molecular testing overall and prior to initiation of first-line therapy has been shown to substantially prolong overall survival compared to those with incomplete or no testing (18).

## 4 Selection of NGS test type

Standard tissue and plasma biopsy assays vary widely in their sensitivity (analytical and diagnostic), specificity, sample requirements (amount and type), turnaround times, and costs (35, 36). Most standard assays (eg, fluorescent *in situ* hybridization, immunohistochemistry, polymerase chain reaction) require significant amounts of sample, have low diagnostic sensitivity, detect only low percentages of *known* alterations for a *single* gene, and are associated with long turnaround times, which translates into treatment delays (35). However, NGS with an appropriate fusion panel enables simultaneous detection of myriad biomarkers and alterations, known or putative, in numerous genes with relatively high analytical and diagnostic accuracy, and from a single biopsy sample (26, 31). This testing modality is in line with the expert recommendation to use “multiplexed genetic sequencing panels over multiple single gene tests” where possible (11).

Though there are multiple types of NGS assays, hybrid capture-based NGS (HC-NGS) testing will provide the most comprehensive, all-inclusive results. This assay provides extensive sequencing information on a broad spectrum of genetic alterations, such as exon/intron mutations, amplifications, rearrangements, fusions, and total tumor mutation burden, in a single assay (31, 37). One limitation introduced by NGS is the difficulty in prioritizing drivers for targeted therapy when multiple drivers are present (31). A list of some commercially available NGS panels for aNSCLC testing can be found in reviews by Cainap et al. (38) and Ionescu et al. (5).

Clinically, NGS can result in vital changes to treatment strategies. One study detected actionable genetic alterations in 76% of clinical tumor specimens using HC-NGS, which was three-times the amount detected by traditional tests (39). In 2017, Rozenblum et al. reported treatment changes, largely replacing chemotherapy, for 37% of patients based on HC-NGS results (31). Their study also demonstrated that the new therapeutic regimens were more effective and less toxic for these patients, thereby offering greater potential for improved quality of life and survival (31). A recent study by Kuang et al. identified at least one genetic variation in more than 80% of upfront NGS-tested samples from patients with newly diagnosed aNSCLC (40). Consequently, 71% of these patients gained access to targeted therapy or became eligible for clinical trial (40).

NGS enables robust detection of gene fusions, which is critical for a precision medicine approach to treating aNSCLC. Addition of an RNA (vs DNA or amplicon) fusion panel to HC-NGS ensures the most accurate detection of rare gene fusions available. DNA/amplicon-based assays cannot detect breakpoints, and rearrangements caused by the fusion event can only be approximated through an imbalance of probes if their design happens to be complementary (41). Therefore, DNA/amplicon-based panels cannot determine whether the fusion is expressed or not (42). On the other hand, while RNA-based panels can be hampered by RNA quality and quantity, analysis at the RNA level captures both known and unknown fusion genes and avoids false-negative results (41). For a more in-depth comparison between DNA/amplicon and RNA gene fusion panels, see Bruno and Fontanini (43).

Guidelines recommend using broad molecular testing modalities to detect biomarkers/alterations in samples with as little as 5% of viable cells and/or 5% of targeted driver alleles (11, 12). Notably, NGS is capable of accurately identifying alterations with an allele frequency as low as 0.2% (17). In addition to fresh plasma, HC-NGS with an RNA fusion panel is best suited for fresh-frozen (vs formalin-fixed paraffin-embedded) tissue samples, though both tissue preservation types can still be used and provide a wealth of information (44, 45). Therefore, it is prudent to run HC-NGS with an RNA fusion panel at the time of diagnosis or resistance/relapse for optimal sample quality and rare fusion detection. Considering the highly sensitive nature of the approach, most NGS reports provide a list of clinical trials for which a patient may be eligible based on their identified biomarker(s)/alteration(s), which will benefit patients who are resistant to or possess molecular alterations that do not yet have an approved and commercially available targeted therapy (35). There are also multiple molecular alteration databases that collect information on genetic alterations and therapies, such as Clinical Interpretation of Variants in Cancer (civicdb.org), My Cancer Genome (mycancergenome.org), and OncoKB (oncokb.org).

## 5 Clinical trial enrollment

Clinical studies pursue novel approaches to combat resistance to current standards of care and acquisition of additional driver alterations with disease progression. A comprehensive list of targeted therapies for rare alterations undergoing testing in clinical trials can be found in a recent review by Michelotti et al. (46). A major hurdle to clinical trials is the enrollment of a sufficient number of patients, particularly for cases of new and/or rare alterations. Comprehensive molecular testing *via* HC-NGS with an RNA fusion panel at local hospital laboratories can help facilitate clinical trial enrollment in two main ways: by 1) pinpointing patients in need by accurate identification of all genetic alterations at a given timepoint, and 2) providing a list of clinical trials for which a patient may be eligible based on their genetic profile as part of the final report.

Clinical trial sponsors have begun actively investing in the development of NGS prescreening and companion diagnostic assays for use in clinical trials, as well as updating existing protocols to include an option for central laboratory prescreening by NGS, if available, that would circumvent the need for locally assessed mutational status in certain cases. There are many actively recruiting NSCLC trials using NGS to study the “liquid biopsy” approach to NSCLC biomarker diagnostics. These studies are using ctDNA NGS to identify biomarkers for risk stratification, predict relapse, and understand mechanisms of resistance. By providing clinicians with training on the necessity, utility, and interpretation of HC-NGS with an RNA fusion panel, they would then be armed with the tools and skills needed to implement comprehensive molecular testing as a standard diagnostic technique, advocate for better insurance coverage, and keep abreast of ongoing or planned trials for rare NSCLC biomarkers/alterations into which they could potentially enroll new patients.

## 6 Patient, family, and caregiver education

Upon diagnosis, patients and their families will experience a plethora of emotions (47, 48), in addition to any physical issues, and likely want to learn as much as possible about NSCLC, what to expect, and the different treatment options available. Anxiety surrounding their diagnosis may trigger a desire to begin therapy as soon as possible. Deficient understanding of their prognosis often leads patients to overestimate their probability of cure, and the majority do not understand their situation well enough to make treatment decisions (49). Providing effective educational resources to patients with NSCLC, their family, and caregivers at the time of diagnosis is an excellent opportunity to not only inform and address questions and concerns but also emphasize the importance of upfront genomic testing for obtaining accurate diagnosis and selecting appropriate therapy. Patients report greater satisfaction when physicians present them with choices in therapeutic decision making (50, 51) and highly rate the necessity for supportive and facilitative dimensions of care (52). Nurses and genetic counselors, in addition to thoracic and community oncologists, all play invaluable roles in providing this education.

Specifically, there is an imminent need to provide effective education to patients with rare NSCLC biomarkers/alterations, as they represent a relatively small fraction of total NSCLC patients and, thus, are underrepresented in the lung cancer community. Within the last decade, NSCLC patients with oncogene driver alterations have organized into online communities with names like ROS1ders and KRAS Kickers, with the goal of addressing the needs of peers with these rare alterations (53, 54). These online communities and organizations provide services such as disease and therapeutic education, support, and even funding for research into new life-saving medicines (53, 54). A recent study by Abbott et al. concluded that groups like these represent powerful resources that improve patient outcomes by enhancing public and patient engagement and meaningful alliances with key stakeholders (53). This study surveyed 465 members from three Facebook-based patient groups in the United Kingdom (EGFR Positive UK, ALK Positive UK, and ROS-1 Support Group) and reported benefits like feeling better prepared, being inspired by other members’ experiences, and being helped with feelings of isolation (53). Only a minority reported struggling when members died and having feelings of increased anxiety (53). This study also confirmed that membership is representative of intended patient populations (53).

Clinician-oriented and/or -driven online resources can also be used for patient and caregiver education purposes. Examples include MedNet (themednet.org), an online resource oriented toward physicians, and CancerGRACE (cancergrace.org), a more patient-focused online resource directed by physicians. Two of the most well-known and comprehensive patient- and caregiver-friendly online educational resources currently available are the American Cancer Society (cancer.org) and American Lung Association (lung.org) (**Table 2**).

**Table 2:** Online non-small cell lung cancer (NSCLC) patient, family, and caregiver education resources.

Resource	Web address	Offerings
General Cancer Resources with NSCLC-specific Information		
American Cancer Society	https://www.cancer.org/	• Disease education • Online support and community
CancerCare	https://www.cancercare.org/	• Disease education • Online support and community
CancerGRACE	https://cancergrace.org/	• Disease education • Online support and community
Cancer.Net	https://www.cancer.net/	• Disease education • Support tips
Macmillan.org.uk	https://www.macmillan.org.uk	• Disease education • Online support and community
The MedNet (physician-only forum)	https://www.themednet.org/	• Physician-only forum
General Cancer Groups		
Cancer Hope Network	https://cancerhopenetwork.org/	• Online support and community
Cancer Support Community	https://www.cancersupportcommunity.org/	• Disease education • Online support and community
Lung Cancer Resources		
American Lung Association	https://www.lung.org/	• Disease education • Online support and community
GO2 Foundation for Lung Cancer	https://go2foundation.org/	• Disease education • Online support and community
Lung Cancer Research Foundation	https://www.lungcancerresearchfoundation.org/	• Disease education
LUNGevity Foundation	https://www.lungevity.org	• Disease education • Online support and community
PIK3CA Testing Navigator	https://www.pik3ca-testing.com/background/	• Disease and testing education
UpToDate.com	https://www.uptodate.com/contents/personalized-genotype-directed-therapy-for-advanced-non-small-cell-lung-cancer/print	• Disease education (overview of personalized, targeted therapy)
WebMD.com	https://www.webmd.com/lung-cancer/story/nsclc-gene-mutations	• Disease education (overview of gene mutations)
Lung Cancer Groups		
ALA Exon 20 Warriors	https://www.inspire.com/groups/exon-20/	• Online support and community
ALA Lung Cancer Survivors	https://www.inspire.com/groups/american-lung-association-lung-cancer-survivors/	• Online support and community
ALK Positive	https://www.alkpositive.org/	• Disease education (limited) • Online support and community
KRAS Kickers	https://www.kraskickers.org/	• Disease education • Online support and community
The ROS1ders	https://www.theros1ders.org/	• Disease education • Online support and community
Social Media Groups (NSCLC Gene Alteration-Specific)		
LUNGevity EGFR Group	https://www.facebook.com/groups/EGFRlung	• Online support and community
EGFR Resisters Lung Cancer Patient Group	https://www.facebook.com/groups/EGFRResisters	• Online support and community
EGFR Exon 18 Group	https://www.facebook.com/groups/1954302018017776/about	• Online support and community
KRAS Kickers	https://www.facebook.com/KRASCancers	• Online support and community
LUNGevity KRAS Blasters	https://www.facebook.com/groups/KRASlung/about	• Online support and community
ALK Positive	https://www.facebook.com/groups/ALKPositive	• Online support and community
KRAS Kickers*	https://www.facebook.com/groups/kraskickers	• Online support and community
The ROS1ders	https://www.facebook.com/TheROS1ders	• Online support and community
BRAF Bombers	https://www.facebook.com/groups/1077213015965166	• Online support and community
MET Crusaders Lung Cancer Patient and Caregiver Support Group	https://www.facebook.com/groups/255429762943996	• Online support and community
MET Lung Cancer Patient Support Group	https://www.facebook.com/groups/575215219879116	• Online support and community
LUNGevity RET Renegades	https://www.facebook.com/groups/RETlung	• Online support and community
RETpositive	https://www.facebook.com/RETpositive	• Online support and community

No social media or other online groups were identified for the following alterations/biomarkers—*NTRK, HER2 (ERBB2), PIK3CA, MEK, FGFR1*, or PD-L1.

*KRAS Kickers mutation-specific subgroups also available on Facebook: G12C, G13D, G12D, G12S, G12V, Q61, and STK11.

Despite the apparent benefits, there is a surprising paucity of peer-reviewed studies or reviews examining the value and role of oncogene-focused patient groups in NSCLC, highlighting a need for the expansion of such efforts. There is also a need for more user-friendly and comprehensive patient-facing educational websites, especially for those with rare forms of NSCLC. Greater availability, knowledge, and access to reputable resources will aid delivery of the most well-rounded care possible, and health care providers are encouraged to steer patients, their families, and caregivers to appropriate patient-friendly educational websites at diagnosis.

## 7 Conclusion

Upfront, mandated comprehensive genomic testing, specifically HC-NGS with an RNA fusion panel, has numerous advantages for patients with aNSCLC at all stages. Major benefits include selection of the most appropriate and personalized treatment, maximization of treatment efficacy, prevention of use of suboptimal/contraindicated therapy, and avoidance of treatment delays, all of which ultimately improve patient outcomes. These advantages should help increase the number of patients who receive complete testing and effective therapy, thereby balancing molecular profiles with prior systemic therapies and patient health. Apart from sample quantity and quality, major barriers to implementing HC-NGS with an RNA fusion panel as part of standard care include lack of equipment/facilities, cost (deficient insurance coverage), delays in obtaining results, which have been exacerbated in recent years due to COVID-19, and insufficient patient and/or clinician knowledge regarding its use and data interpretation (35). While some identified mutations may not be actionable, the information collected may be useful for future analyses and trial enrollment.

In addition to molecular testing, educating patients, their families, and caregivers is key to positive outcomes and survival. Patient education has led to increases in early screening and access to care, especially in high-risk populations (1, 2, 55), and has the potential to increase consent to testing and therapy. Increased patient access to care (eg, insurance coverage) has improved early diagnosis, targeted therapy, and overall survival (1, 2). Cancer survivors must cope with the physical, mental, and economic effects of NSCLC diagnosis and treatment (10). With the advent of social media and increased internet access in general, the volume of patient educational and support resources continues to rise, changing the dynamics of patient care.

Improvements in aNSCLC patient survival continue with the identification of more and more actionable driver alterations and novel targeted therapies. With therapeutic resistance continuing to be a major challenge to aNSCLC treatment, HC-NGS with an RNA fusion panel should become the global diagnostic standard for all disease stages in order to make the most informed therapeutic decision possible.

## Author contributions

All authors contributed to the planning of this manuscript, reviewed at each stage of development, and approved the submitted version.

## References

[1] Siegel RL Miller KD Fuchs HE Jemal A. Cancer statistics, 2022. CA Cancer J Clin (2022) 72(1):7–33. doi: 10.3322/caac.21708 35020204

[2] Liu Y Colditz GA Kozower BD James A Greever-Rice T Schmaltz C. Association of Medicaid expansion under the Patient Protection and Affordable Care Act with non-small cell lung cancer survival. JAMA Oncol (2020) 6(8):1289–90. doi: 10.1001/jamaoncol.2020.1040 PMC7226289 32407435

[3] American Cancer Society. What is lung cancer? (2019). Available at: https://www.cancer.org/cancer/lung-cancer/about/what-is.html (Accessed August 29, 2022).

[4] National Comprehensive Cancer Network. NCCN clinical practice guidelines in oncology (NCCN guidelines®) for non-small cell lung cancer. version 5.2022 (2022). Available at: https://www.nccn.org/guidelines/guidelines-detail?category=1&id=1450.

[5] Ionescu DN Stockley TL Banerji S Couture C Mather CA Xu Z. Consensus recommendations to optimize testing for new targetable alterations in non-small cell lung cancer. Curr Oncol (2022) 29(7):4981–97. doi: 10.3390/curroncol29070396 PMC9318743 35877256

[6] Singh N Temin S Baker S Jr. Blanchard E Brahmer JR Celano P. Therapy for stage IV non-small-cell lung cancer with driver alterations: ASCO living guideline. J Clin Oncol (2022) 40, JCO2200824. doi: 10.1200/JCO.22.00824 35816666

[7] Singh N Temin S Baker S Jr. Blanchard E Brahmer JR Celano P. Therapy for stage IV non-small-cell lung cancer without driver alterations: ASCO living guideline. J Clin Oncol (2022) 40, JCO2200825. doi: 10.1200/JCO.22.00825 35816666

[8] US Food and Drug Administration. Drugs@FDA: FDA-approved drugs. Available at: https://www.accessdata.fda.gov/scripts/cder/daf/index.cfm (Accessed September 26, 2020).

[9] Weaver KE Forsythe LP Reeve BB Alfano CM Rodriguez JL Sabatino SA. Mental and physical health-related quality of life among U.S. cancer survivors: Population estimates from the 2010 national health interview survey. Cancer Epidemiol Biomarkers Prev (2012) 21(11):2108–17. doi: 10.1158/1055-9965.EPI-12-0740 PMC3645867 23112268

[10] Chakravarty D Gao J Phillips S Kundra R Zhang H Wang J. OncoKB: a precision oncology knowledge base. JCO Precis Oncol (2017) 1:1–16. doi: 10.1200/po.17.00011 PMC5586540 28890946

[11] Kalemkerian GP Narula N Kennedy EB Biermann WA Donington J Leighl NB. Molecular testing guideline for the selection of patients with lung cancer for treatment with targeted tyrosine kinase inhibitors: American Society of Clinical Oncology endorsement of the college of American Pathologists/International Association for the Study of Lung/Association for Molecular Pathology clinical practice guideline update. J Clin Oncol (2018) 36(9):911–9. doi: 10.1200/jco.2017.76.7293 29401004

[12] Lindeman NI Cagle PT Aisner DL Arcila ME Beasley MB Bernicker EH. Updated molecular testing guideline for the selection of lung cancer patients for treatment with targeted tyrosine kinase inhibitors: Guideline from the College of American Pathologists, the International Association for the Study of Lung Cancer, and the Association for Molecular Pathology. J Thorac Oncol (2018) 13(3):323–58. doi: 10.1016/j.jtho.2017.12.001 29396253

[13] Gutierrez ME Choi K Lanman RB Licitra EJ Skrzypczak SM Pe Benito R. Genomic profiling of advanced non-small cell lung cancer in community settings: Gaps and opportunities. Clin Lung Cancer (2017) 18(6):651–9. doi: 10.1016/j.cllc.2017.04.004 28479369

[14] Leighl NB Page RD Raymond VM Daniel DB Divers SG Reckamp KL. Clinical utility of comprehensive cell-free DNA analysis to identify genomic biomarkers in patients with newly diagnosed metastatic non-small cell lung cancer. Clin Cancer Res (2019) 25(15):4691–700. doi: 10.1158/1078-0432.CCR-19-0624 30988079

[15] Robert NJ Nwokeji ED Espirito JL Chen L Karhade M Evangelist MC. Biomarker tissue journey among patients (pts) with untreated metastatic non-small cell lung cancer (mNSCLC) in the U.S. Oncology Network community practices. J Clin Oncol (2021) 39(15_suppl):9004. doi: 10.1200/JCO.2021.39.15_suppl.9004

[16] Leighl NB Rekhtman N Biermann WA Huang J Mino-Kenudson M Ramalingam SS. Molecular testing for selection of patients with lung cancer for epidermal growth factor receptor and anaplastic lymphoma kinase tyrosine kinase inhibitors: American Society of Clinical Oncology endorsement of the College of American Pathologists/International Association for the Study of Lung Cancer/Association for Molecular Pathology guideline. J Clin Oncol (2014) 32(32):3673–9. doi: 10.1200/JCO.2014.57.3055 PMC5321089 25311215

[17] Moskalev EA Stohr R Rieker R Hebele S Fuchs F Sirbu H. Increased detection rates of EGFR and KRAS mutations in NSCLC specimens with low tumour cell content by 454 deep sequencing. Virchows Arch (2013) 462(4):409–19. doi: 10.1007/s00428-013-1376-6 PMC3624006 23468066

[18] Aggarwal C Marmarelis ME Hwang W-T Scholes DG McWilliams T Singh AP. Association of comprehensive molecular genotyping and overall survival in patients with advanced non-squamous non-small cell lung cancer. J Clin Oncol (2022) 40(16_suppl):9022. doi: 10.1200/JCO.2022.40.16_suppl.9022

[19] Kris MG Johnson BE Berry LD Kwiatkowski DJ Iafrate AJ Wistuba II. Using multiplexed assays of oncogenic drivers in lung cancers to select targeted drugs. JAMA (2014) 311(19):1998–2006. doi: 10.1001/jama.2014.3741 24846037 PMC4163053

[20] Antonia SJ Villegas A Daniel D Vicente D Murakami S Hui R. Durvalumab after chemoradiotherapy in stage III non-small-cell lung cancer. N Engl J Med (2017) 377(20):1919–29. doi: 10.1056/NEJMoa1709937 28885881

[21] Miura Y Mouri A Kaira K Yamaguchi O Shiono A Hashimoto K. Chemoradiotherapy followed by durvalumab in patients with unresectable advanced non-small cell lung cancer: Management of adverse events. Thorac Cancer (2020) 11(5):1280–7. doi: 10.1111/1759-7714.13394 PMC7180558 32160383

[22] Chan DW Choi HC Lee VH. Treatment-related adverse events of combination EGFR tyrosine kinase inhibitor and immune checkpoint inhibitor in EGFR-mutant advanced non-small cell lung cancer: A systematic review and meta-analysis. Cancers (Basel) (2022) 14(9):2157. doi: 10.3390/cancers14092157 35565285 PMC9102470

[23] Dudnik E Peled N Nechushtan H Wollner M Onn A Agbarya A. BRAF mutant lung cancer: Programmed death ligand 1 expression, tumor mutational burden, microsatellite instability status, and response to immune check-point inhibitors. J Thorac Oncol (2018) 13(8):1128–37. doi: 10.1016/j.jtho.2018.04.024 29723688

[24] Gainor JF Shaw AT Sequist LV Fu X Azzoli CG Piotrowska Z. EGFR mutations and ALK rearrangements are associated with low response rates to PD-1 pathway blockade in non-small cell lung cancer: A retrospective analysis. Clin Cancer Res (2016) 22(18):4585–93. doi: 10.1158/1078-0432.CCR-15-3101 PMC5026567 27225694

[25] Sabari JK Leonardi GC Shu CA Umeton R Montecalvo J Ni A. PD-L1 expression, tumor mutational burden, and response to immunotherapy in patients with MET exon 14 altered lung cancers. Ann Oncol (2018) 29(10):2085–91. doi: 10.1093/annonc/mdy334 PMC6225900 30165371

[26] Villaruz LC Burns TF Ramfidis VS Socinski MA. Personalizing therapy in advanced non-small cell lung cancer. Semin Respir Crit Care Med (2013) 34(6):822–36. doi: 10.1055/s-0033-1358552 PMC4836173 24258572

[27] US Food and Drug Administration. FoundationOne liquid CDx P190032 (2020). Available at: https://www.fda.gov/medical-devices/recently-approved-devices/foundationone-liquid-cdx-p190032 (Accessed August 29, 2022).

[28] US Food and Drug Administration. Guardant360 CDx P200010/S001 (2021). Available at: https://www.fda.gov/medical-devices/recently-approved-devices/guardant360-cdx-p200010s001 (Accessed August 29, 2022).

[29] Zhang M Wu J Zhong W Zhao Z Guo W. Comparative study on the mutation spectrum of tissue DNA and blood ctDNA in patients with non-small cell lung cancer. Transl Cancer Res (2022) 11(5):1245–54. doi: 10.21037/tcr-22-970 PMC9189250 35706796

[30] Page RD Drusbosky LM Dada H Raymond VM Daniel DB Divers SG. Clinical outcomes for plasma-based comprehensive genomic profiling versus standard-of-care tissue testing in advanced non-small cell lung cancer. Clin Lung Cancer (2022) 23(1):72–81. doi: 10.1016/j.cllc.2021.10.001 34782240

[31] Rozenblum AB Ilouze M Dudnik E Dvir A Soussan-Gutman L Geva S. Clinical impact of hybrid capture-based next-generation sequencing on changes in treatment decisions in lung cancer. J Thorac Oncol (2017) 12(2):258–68. doi: 10.1016/j.jtho.2016.10.021 27865871

[32] Cui W Milner-Watts C O’Sullivan H Lyons H Minchom A Bhosle J. Up-front cell-free DNA next generation sequencing improves target identification in UK first line advanced non-small cell lung cancer (NSCLC) patients. Eur J Cancer (2022) 171:44–54. doi: 10.1016/j.ejca.2022.05.012 35704974

[33] Mack PC Banks KC Espenschied CR Burich RA Zill OA Lee CE. Spectrum of driver mutations and clinical impact of circulating tumor DNA analysis in non-small cell lung cancer: Analysis of over 8000 cases. Cancer (2020) 126(14):3219–28. doi: 10.1002/cncr.32876 PMC7383626 32365229

[34] Aggarwal C Davis CW Mick R Thompson JC Ahmed S Jeffries S. Influence of TP53 mutation on survival in patients with advanced EGFR-mutant non-small-cell lung cancer. JCO Precis Oncol (2018) 2018:1–28. doi: 10.1200/PO.18.00107 PMC6372114 30766968

[35] Pennell NA Arcila ME Gandara DR West H. Biomarker testing for patients with advanced non-small cell lung cancer: Real-world issues and tough choices. Am Soc Clin Oncol Educ Book (2019) 39:531–42. doi: 10.1200/EDBK_237863 31099633

[36] Messner DA Al Naber J Koay P Cook-Deegan R Majumder M Javitt G. Barriers to clinical adoption of next generation sequencing: Perspectives of a policy Delphi panel. Appl Transl Genom (2016) 10:19–24. doi: 10.1016/j.atg.2016.05.004 27668172 PMC5025465

[37] Schatz S Falk M Jori B Ramdani HO Schmidt S Willing EM. Integration of tumor mutation burden and PD-L1 testing in routine laboratory diagnostics in non-small cell lung cancer. Cancers (Basel) (2020) 12(6):1685. doi: 10.3390/cancers12061685 32599951 PMC7353063

[38] Cainap C Balacescu O Cainap SS Pop LA. Next generation sequencing technology in lung cancer diagnosis. Biol (Basel) (2021) 10(9):864. doi: 10.3390/biology10090864 PMC8467994 34571741

[39] Frampton GM Fichtenholtz A Otto GA Wang K Downing SR He J. Development and validation of a clinical cancer genomic profiling test based on massively parallel DNA sequencing. Nat Biotechnol (2013) 31(11):1023–31. doi: 10.1038/nbt.2696 PMC5710001 24142049

[40] Kuang S Fung AS Perdrizet KA Chen K Li JJN Le LW. Upfront next generation sequencing in non-small cell lung cancer. Curr Oncol (2022) 29(7):4428–37. doi: 10.3390/curroncol29070352 PMC9319994 35877212

[41] Rodriguez-Antolin C Rosas-Alonso R Cruz P Higuera O Sanchez-Cabrero D Esteban-Rodriguez I. Novel SLC12A2-ROS1 fusion in non-small cell lung cancer with a significant response to crizotinib: The importance of choosing the appropriate next-generation sequencing assay. Oncologist (2021) 26(6):e908–e12. doi: 10.1002/onco.13745 PMC8176992 33682977

[42] Wong D Yip S Sorensen PH. Methods for identifying patients with tropomyosin receptor kinase (TRK) fusion cancer. Pathol Oncol Res (2020) 26(3):1385–99. doi: 10.1007/s12253-019-00685-2 PMC7297824 31256325

[43] Bruno R Fontanini G. Next generation sequencing for gene fusion analysis in lung cancer: a literature review. Diagnostics (Basel) (2020) 10(8):521. doi: 10.3390/diagnostics10080521 32726941 PMC7460167

[44] Martelotto LG Baslan T Kendall J Geyer FC Burke KA Spraggon L. Whole-genome single-cell copy number profiling from formalin-fixed paraffin-embedded samples. Nat Med (2017) 23(3):376–85. doi: 10.1038/nm.4279 PMC5608257 28165479

[45] Gao XH Li J Gong HF Yu GY Liu P Hao LQ. Comparison of fresh frozen tissue with formalin-fixed paraffin-embedded tissue for mutation analysis using a multi-gene panel in patients with colorectal cancer. Front Oncol (2020) 10:310. doi: 10.3389/fonc.2020.00310 32232001 PMC7083147

[46] Michelotti A de Scordilli M Bertoli E De Carlo E Del Conte A Bearz A. NSCLC as the paradigm of precision medicine at its finest: The rise of new druggable molecular targets for advanced disease. Int J Mol Sci (2022) 23(12):6748. doi: 10.3390/ijms23126748 35743191 PMC9223783

[47] Chapple A Ziebland S McPherson A. Stigma, shame, and blame experienced by patients with lung cancer: Qualitative study. BMJ (2004) 328(7454):1470. doi: 10.1136/bmj.38111.639734.7C 15194599 PMC428516

[48] Uchitomi Y Mikami I Nagai K Nishiwaki Y Akechi T Okamura H. Depression and psychological distress in patients during the year after curative resection of non-small-cell lung cancer. J Clin Oncol (2003) 21(1):69–77. doi: 10.1200/JCO.2003.12.139 12506173

[49] Quirt CF Mackillop WJ Ginsburg AD Sheldon L Brundage M Dixon P. Do doctors know when their patients don’t? A survey of doctor-patient communication in lung cancer. Lung Cancer (1997) 18(1):1–20. doi: 10.1016/s0169-5002(97)00048-2 9268944

[50] Leighl N Gattellari M Butow P Brown R Tattersall MH. Discussing adjuvant cancer therapy. J Clin Oncol (2001) 19(6):1768–78. doi: 10.1200/JCO.2001.19.6.1768 11251008

[51] Kris MG Gaspar LE Chaft JE Kennedy EB Azzoli CG Ellis PM. Adjuvant systemic therapy and adjuvant radiation therapy for stage I to IIIA completely resected non-small-cell lung cancers: American Society of Clinical Oncology/Cancer Care Ontario clinical practice guideline update. J Clin Oncol (2017) 35(25):2960–74. doi: 10.1200/JCO.2017.72.4401 28437162

[52] Parker PA Baile WF de Moor C Lenzi R Kudelka AP Cohen L. Breaking bad news about cancer: Patients’ preferences for communication. J Clin Oncol (2001) 19(7):2049–56. doi: 10.1200/JCO.2001.19.7.2049 11283138

[53] Abbott J Beattie K Montague D. The role of UK oncogene-focussed patient groups in supporting and educating patients with oncogene-driven NSCLC: Results from a patient-devised survey. Oncol Ther (2021) 9(1):187–93. doi: 10.1007/s40487-021-00145-5 PMC8140168 33677715

[54] Dolgin E. Oncogene-specific advocacy groups bring a patient-centric perspective to studies of lung cancer. Nature (2020) 587(7834):S16–S7. doi: 10.1038/d41586-020-03150-2 33208972

[55] Fedewa SA Kazerooni EA Studts JL Smith RA Bandi P Sauer AG. State variation in low-dose computed tomography scanning for lung cancer screening in the United States. J Natl Cancer Inst (2021) 113(8):1044–52. doi: 10.1093/jnci/djaa170 PMC8328984 33176362

